# Mitochondrial dysfunction in EEG: high-beta specific metabolic/oxidative coupling with lactate in mania-predominant polarity

**DOI:** 10.3389/fpsyt.2026.1727711

**Published:** 2026-04-20

**Authors:** Sermin Kesebir, Rüştü Murat Demirer

**Affiliations:** 1 Department of Psychiatry, School of Medicine, Üsküdar University, Istanbul, Türkiye; 2 Natural and Engineering Sciences, Üsküdar University, Istanbul, Türkiye

**Keywords:** bipolar disorder, EEG, lactate, mania predominant polarity, metabolic syndrome, mitochondrial dysfunction

## Abstract

Psychiatric diseases are progressively recognized as disruptions in brain dynamics that can be measured using electrophysiological and metabolic indicators. Conventional EEG investigations utilizing power spectra or standard entropy metrics (sample entropy, multiscale entropy, permutation entropy) have shown diagnostic significance but are constrained in their ability to capture higher-order relationships. This work combines psychiatric ideas with sophisticated mathematical methods to discover new indicators of modified brain complexity. From an engineering standpoint, we provide entropy-doubling, an information-theoretic approach utilized for EEG signals for the first time. In contrast to conventional entropy measures, entropy-doubling assesses the evolution of informational complexity under distributional convolution, hence uncovering redundancy and structural scaling characteristics that are not discernible through existing metrics. We associate these metrics with biochemical markers, such as lactate, to investigate potential metabolic coupling of brain processes in bipolar disorder. It has the characteristics of a biphasic energy dysregulation. The aim of this study is to investigate the correlation between electrophysiological brain dynamics, as quantified by entropy doubling and Ruzsa distance measures derived from EEG signals, and lactate levels, in patients with bipolar disorder with mania-predominant polarity. According to our results, high-beta amplitude entropy provides the strongest and most consistent correlations with lactate. This combined framework of psychiatry and biomedical engineering provides a novel, interpretable pathway toward quantitative biomarkers in psychiatric research.

## Introduction

Lactate is increasingly recognized not just as a glycolytic by-product but as a key oxidative substrate and signaling “lactormone” for neurons under physiological and cognitive challenge (1–4). The astrocyte–neuron lactate shuttle (ANLS) model posits that astrocytes produce lactate that is shuttled to neurons via MCT transporters to fuel mitochondrial metabolism. In line with this, functional MRI studies have consistently demonstrated that lactate levels rise during intense neural activation and that EEG high-frequency power often co-varies with extracellular lactate dynamics (5).

The brain’s high-energy activity requires glucose as fuel (6). Lactate is an alternative fuel for its low-energy activity. Lactate has been suggested as a peripheral biomarker for mitochondrial dysfunction (7). In bipolar disorder, lactate was found to be elevated in the brain in six studies and in CSF in two studies using MRS. Peripheral measurements showed two positive and two negative results. Two separate studies showed that elevated serum lactate was associated with depressive episodes and reversible with lithium (8, 9).

The predominant polarity in BD may be differentially associated with several clinical correlates (10). The predominant polarity as a course specifier for BD proposed a threshold of at least two-thirds of lifetime depressive episodes for the definition of a depressive-predominant polarity, while at least two-thirds of past episodes would fulfill the criteria for mania/hypomania and define a manic-predominant polarity. The concept of a polarity index (PI) has been recently proposed as an index of the antimanic versus antidepressive efficacy of various maintenance treatments for BD.

The aim of this study is to investigate the correlation between electrophysiological brain dynamics, as quantified by entropy doubling and Ruzsa distance measures derived from EEG signals, and peripheral metabolic markers, specifically lactate levels, in bipolar disorder with mania predominant polarity. Bipolar disorder has the characteristics of a biphasic energy dysregulation (11–16). Mitochondrial dysfunction is an important area of ​​research in the etiology of this neuroprogressive disorder. By focusing on correlation patterns across frequency bands and electrode sites, this study seeks to identify whether complex information-theoretic properties of EEG activity are systematically associated with bioenergetic processes, thereby providing a novel framework to link cortical signal organization with systemic metabolism.

## Methods

### Work motivation

The motivation for this study arises from both clinical and methodological considerations:

- Clinical perspective: There is a pressing need for quantitative and objective biomarkers that capture the electrophysiological and metabolic alterations underlying psychiatric disorders. EEG offers a non-invasive window into brain dynamics, and its integration with biochemical measures such as lactate provides a unique opportunity to characterize the metabolic–neural interface.
- Methodological perspective: Traditional methods such as power spectral density, sample entropy, multiscale entropy, permutation entropy, and fractal dimensions have been shown to be effective in differentiating mental conditions. Nonetheless, these methods predominantly measure unpredictability or the scaling of local patterns and may inadequately identify redundancy structures and higher-order connections in brain signals. We provide entropy-doubling as an innovative analytical instrument for EEG to overcome this constraint. In contrast to conventional entropy measures, entropy-doubling assesses the evolution of information complexity through the convolution of distributions, so revealing self-similarity, structured redundancy, and distributional scaling characteristics that normal methods overlook.
- Translational perspective: By systematically applying entropy-doubling alongside established complexity measures, and by correlating these results with metabolic markers, we aim to establish an integrated, interpretable biomarker framework for psychiatric disorders. To our knowledge, this represents the first application of entropy-doubling to EEG data, thus extending the methodological repertoire available for computational psychiatry and bridging biomedical engineering with clinical neuroscience.

### Assumptions

Our cross-disciplinary approach relies on several key assumptions:

1. EEG as proxy: Despite limited spatial resolution, EEG carries sufficient temporal and spectral information to characterize psychiatric brain dynamics.
2. Stationarity of short epochs: EEG is globally non-stationary, but short (e.g., 2-second) epochs can approximate stationarity for entropy and additive measures.
3. Complexity as biomarker: Metrics such as entropy-doubling, multiscale entropy, and additive energy capture physiologically relevant signal structures that correlate with psychiatric states.
4. Metabolic coupling: Variations in lactate is assumed to modulate neural complexity, making them meaningful covariates in EEG analyses.
5. Mathematical transferability: Abstract constructs like Ruzsa distance and additive combinatorics, after discretization, remain valid when applied to real-valued EEG signals.
6. Interpretability: These mathematically defined metrics can be contextualized within psychiatry when interpreted collaboratively between engineering and clinical expertise.

### Sample

16 patients diagnosed with Bipolar Disorder Type 1 according to DSM-5 were consecutively evaluated during their remission period when they presented to our outpatient unit for routine check-ups in this study. The requirement of being euthymic for at least 8 weeks was met with HDRS and YMRS applications.

The study inclusion criterion was a history of mania predominant polarity. The predominant polarity as a course specifier for BD proposed a threshold of at least two-thirds of lifetime depressive episodes for the definition of a depressive-predominant polarity, while at least two-thirds of past episodes would fulfill the criteria for mania/hypomania and define a manic-predominant polarity. Predominant polarity was determined on the lifechart by the patient and at least one family member, within the scope of the inclusion criteria for this study.

Metabolic syndrome was an exclusion criterion: i) hypertension, ii) obesity, iii) dyslipidemia, iv) insulin resistance and diabetes, and v) elevated uric acid. Multivariate regression analyses have shown that lactate is associated with triglycerides, blood glucose, and systolic and diastolic blood pressure (17, 18). Mitochondrial markers vary in patients with metabolic syndrome, with normal and elevated lactate levels. Another exclusion criterion was the use of typical antipsychotics, as a possible elevation in creatine kinase would affect lactate levels (19). All patients were using lithium and/or an antiepileptic agent as a mood stabilizer.

Our previous study was performed on remission of bipolar patients without any distinction of subgroups such as polarity or the like titled ‘‘Serum lactate and LDH are related with Teta and Gama activities in Bipolar Disorder: A Band-Specific Metabolic Coupling’’ (20). The current submission is focusing on mania-predominant polarity. The sample in our current study is an independent group from the previous one.

Ethics committee approval was obtained from our university board, and our university project-based research fund was used as the source. Serum lactate levels were measured, and EEG recordings were performed in 16 subjects who gave informed consent. Blood sampling and EEG recording were performed sequentially, following clinical evaluation. This article did not receive any specific grant from funding agencies in the public, commercial, or not-for-profit sectors.

All EEG data was recorded in a quiet, subtly lit room, in sitting position, with eyes closed. Nineteen scalp electrodes were placed according to the 10–20 system. Linked mastoid electrodes (A1–A2) were used for reference. EEG was recorded at a sample rate of 125 samples/s. Recording time was 3 min. Impedances for each electrode referring channels were kept below 30 kΩ. EEG processed offline for artifact rejection. A high pass filter was applied at 0.1 Hz and a low pass filter was applied at 70 Hz. The Hilbert-based Entropy Doubling approach was used to process the analytical signals for each EEG channel.

This study is following the same methodology explained in mathematical approach. Unlike our previous work, narrow-band decomposition was used, such as low and high beta and low and high gamma. Low beta was recorded as 12–20 Hz, high beta as 20–30 Hz, low gamma as 30–40 Hz, and high gamma as 40–70 Hz. Unlike our previous study, phase entropy was examined in addition to amplitude entropy.

### Mathematical approach

We employed entropy-based metrics recently formalized in additive combinatorics to quantify hidden structural redundancy in EEG signals. We go through the steps, histogram definition, convolution, entropy-doubling equations and zero-phase guarantee.

#### EEG preprocessing and band definition

Raw EEG recordings were imported from EDF format and preprocessed using the FieldTrip toolbox (21). Data were sampled at 125 Hz, which was the acquisition rate for all patients. Signals were re-referenced to the common average reference after removal of non-EEG channels.

Continuous data were segmented into non-overlapping epochs of 2s (250 samples per epoch). Each epoch was then bandpass filtered into canonical frequency bands. Filters were designed as linear-phase finite impulse response (FIR) filters using the windowed sinc method, implemented with zero-phase forward-backward application (filtfilt command in MATLAB/FieldTrip) to avoid phase distortion (22). Stopband attenuation was at least 40 dB, with transition widths of ∼10% of the passband edge frequency.

#### Entropy doubling

Entropy doubling features were computed by adapting the entropic doubling constant (Equation 1):

(1) $$\sigma_{\text{ent}}[X]=\text{exp}(H(X_{1}+X_{2})-H(X))$$

where $X_{1},X_{2}$ are independent copies of the discretized EEG signal and H(·) denotes Shannon entropy, following the formulation (23, 24). For each EEG epoch, the empirical distribution $p_{X}$ (histogram) is estimated from amplitude or phase samples, and entropy is computed for both the original and doubled distributions. This procedure highlights repetitions and hidden organizational patterns beyond random variability.

### Entropic ruzsa distance

We further evaluated the entropic Ruzsa distance based on the same classical Shannon entropy (Equation 2).

(2) $$d_{\text{ent}}(X,Y)=H(X^{\prime }-Y^{\prime })-\frac{1}{2}H(X^{\prime })-\frac{1}{2}H(Y^{\prime })$$

where $X^{\prime },Y^{\prime }$ are independent copies of X,Y, respectively. In our adaptation, EEG epochs were discretized into empirical probability distributions, and pairwise entropic distances were computed between channels in a 10–20 electrode system. This measure captures the degree of independence or redundancy among brain regions.

### Theoretical link between entropy doubling and entropic ruzsa distance

The two measures are mathematically related by (Equation 3):

(3) $$\sigma_{\text{ent}}[X]=\text{exp}(d_{\text{ent}}(X,-X))$$

which provides a unified framework for assessing both within-channel structural complexity and cross-channel dependence in EEG dynamics.

### Smoothing window

We stabilize empirical distributions within each epoch $E_{k}=[t_{k},t_{k}+T)$, apply a symmetric kernel $g_{\tau }(t)$ (Equations 10a, b):

(10-a) $${\tilde{a}}_{b}(t)=(g_{\tau }*a_{b})(t)$$

(10-b) $${\tilde{\phi }}_{b}(t)=\text{unwrap}((g_{\tau }*\phi_{b})(t))$$

### Vectorization operator

For each epoch $E_{k}$ with sample grid ${\left\{t_{j}\right\}}_{j=1}^{N}$

(11) $$\begin{array}{l} a_{b,k}={\left\{{\tilde{a}}_{b}(t_{j})\right\}}_{j=1}^{N}\in {\mathbb{R}}^{N}\\ \phi_{b,k}={\left\{{\tilde{\phi }}_{b}(t_{j})\right\}}_{j=1}^{N}\in {\mathbb{R}}^{N} \end{array}$$

where ${\left\{t_{j}\right\}}_{j=1}^{N}$ are the discrete sampling points inside epoch $E_{k}$.

• $a_{b,k}$ is simply the vector of amplitude samples in epoch
• $E_{k}$. $\phi_{b,k}$ is the vector of phase samples in epoch $E_{k}$.

Independent-copy property is guaranteed on only circular shifts. We generate two approximately independent surrogates by applying circular shifts to the amplitude and phase vectors within each epoch (Equation 11).

• Epoch integer index *k*: each trial or segment is labeled by *k*.
• Sample index n∈{0,…,N−1}: denotes the discrete time sample inside epoch $E_{k}$. For example 2 sec. epoch corresponds to N=250 samples in the case of sampling frequency of $f_{s}=125\;Hz.$• Shift amount s is an integer (uniformly chosen in [1,N−1]) representing how many samples we circularly rotate.

Formally, for the amplitude vector $a_{b,k}[n]$ (Equation 12a):

(12-a) $$\left(T_{s}a_{b,k})[n]=a_{b,k}[(n-s)\text{mod}N\right]$$

and for the phase vector $\phi_{b,k}[n]$ (Equation 12b):

(12-b) $$\left(T_{s}\phi_{b,k})[n]=\phi_{b,k}[(n-s)\text{mod}N\right]$$

We define surrogates (Equations 13a, b):

(13-a) $$X_{1}^{\left(a\right)}=T_{s_{1}}a_{b,k}$$

(13-b) $$X_{2}^{\left(a\right)}=T_{s_{2}}a_{b,k}$$

We can write the surrogates similar analogously for phase. This avoids block-bootstrap operators. The circular shift ensures all samples are preserved, only permuted. Using two different random shifts creates two copies of the same distribution that are decorrelated enough to act as independent samples. This procedure avoids block-bootstrap resampling (no cutting/rejoining of time series) while preserving amplitude and phase statistics within each 2-sec epoch.

### Distribution-level convolution

Let X denote a vector of values from one epoch, either amplitude samples ${\left\{a_{b,k}[n]\right\}}_{n=1}^{N}$ or phase samples ${\left\{\varphi_{b,k}[n]\right\}}_{n=1}^{N}$. To estimate its probability distribution, we construct an empirical histogram with $n_{\text{Bins }}=50$ equal-width bins (linear for amplitude, circular for phase). The normalized histogram yields the discrete distribution (Equation 14).

(14) $$p_{X}[j]=\frac{1}{N}\#\{n:X[n]\in {\text{bin}}_{j}\},\;j=1,\ldots ,n_{\text{Bins }}$$

This $p_{X}$ represents the probability distribution of a single surrogate copy of the data. Since $X_{1}$ and $X_{2}$ are generated by independent circular shifts of the same signal, their distributions are identical, both equal to $p_{X}$. The distribution of their sum is then defined by discrete convolution (Equation 15):

(15) $$\begin{array}{c} p_{S}=p_{X}*p_{X}\\ p_{S}\left[k\right]=\sum_{i}p_{X}\left[i\right]p_{X}\left[k-i\right] \end{array}$$

Entropy is then estimated for both $p_{X}$ and $p_{S}$ using the Shannon estimator, yielding the entropy-doubling statistic. This step follows the entropy-doubling framework (25, 26).

### Entropy-doubling estimators

With equal-width bins [*n* Bins =50 for amplitude; circular bins for phase (Equation 16)]:

(16) $$\begin{array}{l} {\hat{\sigma }}_{ent}[a_{b}]=\text{exp}(\hat{H}(X_{1}^{\left(a\right)}+X_{2}^{\left(a\right)})-\hat{H}(X^{\left(a\right)})),\\ {\hat{\sigma }}_{ent}[\phi_{b}]=\text{exp}(\hat{H}(X_{1}^{\left(\phi \right)}+X_{2}^{\left(\phi \right)})-\hat{H}(X^{\left(\phi \right)})). \end{array}$$

Here, $\hat{H}(\cdot )$ is Shannon entropy from empirical histograms ( $p_{X}$, $p_{S}$), as in Dembo et al. (27).

Zero-phase guarantee must be provided for guaranteeing the entropy doubling. Since $h_{b}$ is linear-phase (applied zero-phase) and $g_{\tau }$ is symmetric, the sequence (Equation 17)

(17) $$x\to x_{b}\to z_{b}\to (a_{b},\phi_{b})\to ({\tilde{a}}_{b},{\tilde{\phi }}_{b})\to (a_{b,k},\phi_{b,k})$$

introduces no phase distortion (28).

## Results

The mean age of the 11 female and 5 male cases was 33.7 ± 12.5 years, and the disease duration was 14.6 ± 9.5 years. The average lactate levels were calculated to be 1.8 ± 0.3 mmol/l.

**Figure 1:**
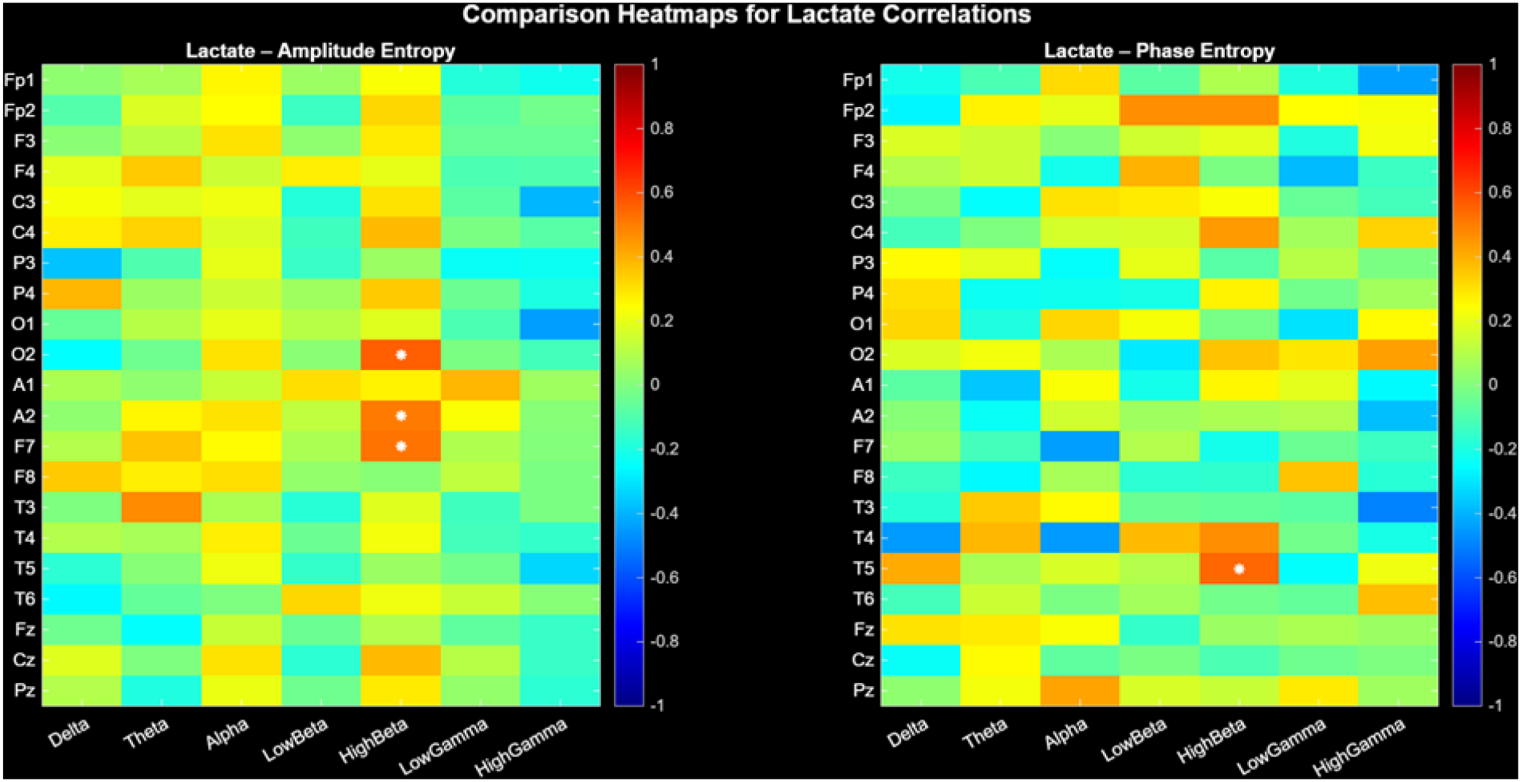
Lactate correlations computed using Spearman correlation on EEG amplitude and phase entropy metrics. Each heatmap cell represents correlation coefficient (red=positive, blue=negative), with statistically significant correlations (p<0.05) marked by white asterisks. The barcode-like vertical stripes in the heatmaps correspond to frequency-specific effects: each frequency column shows a distinct pattern of correlations across electrodes. Strong red or blue ‘bars’ indicate band-driven effects, confirming that metabolic coupling is frequency-specific rather than diffuse. Confidence intervals (CIs) provide precision context. Narrow CI ranges indicate reliable correlation estimates, while wider ranges warrant cautious interpretation.

Energy dysregulation includes high-beta frequency bands in relation to lactate (**Figure 1**). Each heatmap cell represents correlation coefficient (red=positive, blue=negative), with statistically significant correlations (p<0.05) marked by white asterisks. The barcode-like vertical stripes in the heatmaps correspond to frequency-specific effects: each frequency column shows a distinct pattern of correlations across electrodes. Strong red or blue ‘bars’ indicate band-driven effects, confirming that metabolic coupling is frequency-specific rather than diffuse. Confidence intervals (CIs) provide precision context. Narrow CI ranges indicate reliable correlation estimates, while wider ranges warrant cautious interpretation.

For Lactate, significant amplitude entropy correlations emerged high-beta at F7 (r= 0.518, p= 0.042), A2 (r= 0.506, p= 0.048) and O2 (r= 0.556, p = 0.028). T5 high-beta phase entropy correlated with lactate (r= 0.544, p= 0.032), (**Table 1**). The false discovery rate (FDR) was computed using methodology described by Benjamini and Hochberg (29). Significant results were determined based on an FDR-adjusted *p*-value of ≤ 0.05. Correlation at A2 was non-significant according to FDR-adjusted *p*-value.

**Table 1:** Correlations table.

r, p (FDR p)	Lactate
F7 High beta	0.518, 0.042
A2 High beta	0.506, 0.048 (NS)
O2 High beta	0.556, 0.028
T5 High beta	0.544, 0.032

Amplitude Entropy maintained significant localized effects, particularly in high-beta. Phase Entropy exhibited diminished strength and geographic dispersion, indicating that phase timing disruptions arise after or under increased metabolic stress.

## Discussion

Our results demonstrate that high-beta amplitude entropy provides the strongest and most consistent correlations with both lactate levels, particularly at F7 and O2. Phase entropy correlations were weaker in this sample. The probability is this, amplitude entropy doubling captures irregularity of envelope fluctuations sensitive to disrupted or inefficient oscillatory regulation under metabolic stress whereas phase entropy doubling remained relatively unremarkable, implying phase coherence may be preserved until more severe metabolic disruption occurs. On the other hand, phase entropy correlations were more localized. Narrow-band decomposition was essential to uncover these associations.

The correlations between lactate and entropy-based brain dynamics were found in our previous study, which did not consider dominant polarity, at the F7 and O2 electrodes. Regarding this localization, which we will position anterior-posteriorly, we will emphasize it and refrain from commenting at this stage. Together, these findings support the use of high-beta amplitude entropy as a candidate EEG biomarker of metabolic state in mania dominance. With further validation in larger cohorts and multimodal contexts, it may aid in tracking neuroenergetic load, cognitive impairment, and systemic metabolic dysfunctions.

In our previous study, we demonstrated a linear relationship between serum lactate levels and theta activity and a strong negative relationship between gamma activity in bipolar patients during remission (20). Accordingly, we suggest that band-specific metabolic coupling is different between patients with mania predominant polarity and those with depression predominant polarity. The findings of this study support the validity and reliability of the predominant polarity definition.

In the distinction between state and trait, the manic-predominant polarity matches the hyperthymic temperament, which has been proposed as a marker of resilience for bipolar disorder (30–34). In our previous study, a strong inverse relationship between lactate and gamma activity was associated with cognitive impairment (20). The most important finding of this study is that impairment in high-frequency bands is not observed in cases with a manic-predominant polarity. Future studies must combine the relationship between metabolic/oxidative stress and entropy-based brain dynamics with measures that assess cognitive function. Indeed, animal studies revealing high lactate levels in the brain have suggested that this may be a transdiagnostic endophenotype characterized by cognitive impairment in bipolar disorder, as well as schizophrenia, autism, epilepsy, and Alzheimer’s disease. A common feature of 5 studies involving 2294 animals is the association between high lactate levels and poor working memory performance (35).

Correlation cannot confirm causation. EEG captures millisecond-scale activity, while lactate related changes are slower, which future simultaneous fMRI-EEG protocols will clarify temporal coupling. Another significant challenge is the lack of EMG control over noise in the high-frequency bands. The small sample size and the inclusion of lithium and anticonvulsants in prophylactic treatment are other limitations of this study. However, we must reiterate that the use of antipsychotics, which would have created more problems, has been excluded (17–19). For multiple comparisons, an FDR (Force Diffuser Ratio) correction was applied (29).

In this study, we asked a simple, yet valid and important question. This is true because we found highly significant correlations. This is important because it was investigated for the first time. In conclusion, these findings resonate with emerging neuroscience insights that link global oscillatory energy patterns, rather than signal irregularity alone, to metabolic and bioenergetic modulation in the brain. Given lactate’s dual role as fuel and signaling molecule involved in neuroplasticity, angiogenesis, and gene regulation, the EEG–metabolite associations may reflect integrated energetic and neuroprotective processes, potentially relevant in cognitive health, resilience to stress, and neurodegenerative risk (5).

Repeated findings have shown that it significantly supports synaptic plasticity (6). High lactate levels are present in conditions such as ischemic and traumatic brain injury, and sepsis. The interactions discussed thus far support the necessity of therapeutic infusion of exogenous lactate in cases of ischemic and traumatic brain injury, and sepsis. The energy dysregulation associated with a mood episode can be likened to the relationship between lactate and glucose utilization/oxidative phosphorylation and glycolysis, or even a Warburg effect as in cancer. At this point, the question of whether Ringer’s lactate may be a mood stabilizer can be considered a valid and necessary one. At this point, we propose that in mood disorders with predominant polarity mania, glucose metabolism is less impaired and lactate metabolism is more functional, which is the subject of future studies.

## Author’s note

This research is related to a previous study by the same authors titled ‘‘Serum lactate and LDH are related with Teta and Gama activities in Bipolar Disorder: A Band-Specific Metabolic Coupling’’ (20). The previous study was performed on remission of bipolar patients without any distinction of subgroups such as polarity or the like. The current submission is focusing on mania-predominant polarity as an unipolar mania. The sample in our current study is an independent group from the previous one. The study is following the same methodology explained in mathematical approach. Unlike our previous work, narrow-band decomposition was used, such as low and high beta and low and high gamma. Narrow-band decomposition was essential to uncover our associations. Unlike our previous study, phase entropy was examined in addition to amplitude entropy.

## Data Availability

The raw data supporting the conclusions of this article will be made available by the authors, without undue reservation.
